# MEDICAL MARIJUANA LAWS AND OUT OF POCKET EXPENSES AT END OF LIFE

**DOI:** 10.1093/geroni/igac059.394

**Published:** 2022-12-20

**Authors:** Divya Bhagianadh, Kanika Arora

**Affiliations:** University of Iowa, Iowa city, Iowa, United States; University of Iowa, Iowa city, Iowa, United States

## Abstract

Resource intensive and costly End-of-Life (EOL) care is a significant healthcare policy concern in the U.S. In addition to the high Medicare spending, out of pocket (OOP) expenses are also high during EOL adding considerable stress during terminal days. The Medical Marijuana Laws (MMLs) is a significant policy in this context. Previous studies have shown increased use of MM among older adults, better pain management, influence on site of death as well as evidence of reduction in Medicare expenditure on drugs in states with MMLs. In this study, we explore the association between MML and OOP expenses during EOL using data from the Health and Retirement Study exit and core interviews from 1995 to 2018. We use a difference in differences (DD) and event study models to examine this question. We find evidence of increased OOP expenses on drugs and doctor visits with the effects concentrated among the early implementing states, among decedents who are White and among cancer patients. Despite its growing acceptance in palliative medicine, affordability of MM could pose a significant hurdle to terminally ill patients especially since MM and related costs are not covered by insurance.

